# Exploring the Notion of Literacy Within Physical Literacy: A Discussion Paper

**DOI:** 10.3389/fspor.2022.853247

**Published:** 2022-05-03

**Authors:** Elizabeth J. Durden-Myers, Gillian Bartle, Margaret E. Whitehead, Karamjeet K. Dhillon

**Affiliations:** ^1^The School of Education, Bath Spa University, Bath, United Kingdom; ^2^The School of Education and Humanities, The University of Gloucestershire, Cheltenham, United Kingdom; ^3^School of Education and Social Work, University of Dundee, Dundee, United Kingdom; ^4^Faculty of Social Sciences, University of Stirling, Stirling, United Kingdom; ^5^Faculty of Education and Sport, The University of Bedfordshire, Bedford, United Kingdom; ^6^Social Research, Boost Innovations, Windsor, ON, Canada

**Keywords:** literacy, literate, illiterate, embodiment, physical activity, physical education, physical literacy, flourishment

## Abstract

The concept of physical literacy is continuing to gain traction internationally. This increasing interest has also given rise to concerns about the use, interpretation and meaning of the term “literacy” within the context of physical literacy. This paper explores the development of the terms literate, illiterate, literacy, and illiteracy identifying their historical origin and contemporary meaning. This provides the backdrop to explore the use of the term literacy within the context of physical literacy. In the final part of this introductory section the recent popularity of the literacies movement is explored. Our discussion identifies key intersections and areas of tension associated with the use, interpretation and meaning of literacy in the context of physical literacy. We adopt Whitehead's philosophy of physical literacy and discussion is informed further by Derrida's notion of *differance*, and Barad's challenge to singular representations of concepts. Once harnessing these concepts, we reach a juncture of an in-between space; entry points of nonidentity (sameness) and points where multiple effects of difference are created. Key discussion topics include: discourse, language and interpretations of literacy; in/tangibility of literacy; capturing literacy; literacy as a process or a product; connotations of the terms literate and illiterate; neoliberalism and literacy and finally literacy as learning. We believe that when understood as the productive and meaningful interaction with/in/through the world, literacy is still the appropriate term within the context of physical literacy. Our discussion leads us to conclude that as embodied individuals, physical literacy is often the literacy through which other literacies have to pass. Through physical activity individuals can not only nurture their own physical literacy but also contribute toward a global or holistic literacy that helps us navigate, connect and make sense of ourselves, others and the world around us. However, the paper acknowledges that this meaning is not always grasped with the historical understanding of literacy as well as it's translations into other languages presenting challenges in articulating the intended use, meaning and connotations of the contemporary understanding of physical literacy.

## Introduction

In 2005, the United Nations Educational, Scientific and Cultural Organisation's (UNESCO) released *Education for All: Literacy for Life* [United Nations Educational, Scientific and Cultural Organisation (UNESCO), 2005]. This position paper provides a background to the definition and historical development of the term literacy. It also identifies aspects evident in contemporary understandings of literacy as being more than just reading and writing, describing it more broadly as the ability to identify, understand, interpret, create, communicate and compute, in varying contexts [Freire and Macedo, 1987; Stevens-Smith, 2016; United Nations Educational, Scientific and Cultural Organisation (UNESCO), 2019].

Literacy is typically viewed as falling somewhere on a continuum ranging from a set of skills to a basis for rational and ethical action (Bailey et al., 1998). For example, Freire and Macedo (1987) refer to literacy as the ability to not only read the word but to also read the world. As these descriptions suggest, literacy is broader than just the acquisition of knowledge and understanding of content. Further, United Nations Educational, Scientific and Cultural Organisation (UNESCO) (2005, p. 14) suggest that literacy enables individuals to assume both a personal and social responsibility to use the knowledge gained from lived experiences in ethical and just ways and “*participate fully in their community and wider society*.” Being literate includes the use of critical and creative thinking skills and/or processes, conveying information through various forms of communication and applying knowledge and skills to make connections within and between various contexts [United Nations Educational, Scientific and Cultural Organisation (UNESCO), 2019].

Whilst the latter discussion on literacy and forms of communication shows broader contemporary understandings of what is meant by the term literacy, there remain historical interpretations and understandings of the term, which we intend to explore. Therefore, this paper seeks to encourage further discussion in two main areas; first, the understanding(s) of literacy and second understanding(s) in the context of physical literacy. The contribution this paper makes is to explore historical and contemporary uses of the term “literacy” with the view to embedding a richer notion of it before it allies with “physical.” We identify and discuss key intersections and areas of tension which have emerged in relation to literacy as applied to physical literacy. Whilst providing insight into the intersections as we identify them, we draw from Derrida's (1973) notion of *differance*, and Barad's (2013) challenge to singular representations of concepts. Thus, whilst being mindful of Whitehead's (2010) philosophy of physical literacy, we invite multiple understandings and practisings of the concept, embracing inclusivity, and the existential and phonological roots of physical literacy. Tensions include multiple interpretations of literacy; in/tangibility of literacy; capturing literacy; literacy as a process or a product; connotations of the terms literate and illiterate; neoliberalism and literacy, and literacy as learning.

### The Historical Origins of the Terms Literate, Literacy, Illiterate and Illiteracy

The Online Etymology Dictionary (2021) cites literate as originating from the Latin *literatus*/*litteratus* meaning “educated, learned, who knows the letters.” Its origin can be traced back to the early 15th century (15c.), with descriptions including “educated, instructed or having knowledge of letters.” By the late 18c. the term was frequently associated with being “acquainted with literature” and by 1894 also used as a noun, to mean “one who can read and write.” Literacy derives from literate, and also emerged in the late 18c. being described in 1883 as the “ability to read and write.” Similarly, the origins of “illiterate” can be traced back to the early 15c. with its meaning described as being “uneducated, unable to read and write.” Illiterate comes from the Latin *illiteratus* meaning “unlearned, unlettered, ignorant; without culture, inelegant.” The origin of Illiteracy can be traced back to the mid 16c. deriving from illiterate to mean the “inability to read and write.”

The use of these terms has naturally evolved and changed over time. Historically, literacy has predominantly been used in relation to English literacy, language and the ability to read and write. Prior to the late 20th century there were very few uses of the term “literacy” beyond its reference to the skills of reading and writing. However, in the last 20 years a range of areas have adopted the suffix of literacy. The number of areas adopting the term is now probably over two dozen, ranging from emotional literacy to food literacy and from environmental literacy to political literacy. In a brief literature search the following literacies were identified: Mathematical (Jablonka and Niss, 2014), Leisure (Ayyildiz-Durhan, 2020), Historical (Maposa and Wassermann, 2009), Musical (Csíkos and Dohány, 2016), Geographical (Kamil et al., 2020), Dance (Jones, 2014), Aesthetic (Greene, 1977), Health, Nutrition and Food (Velardo, 2015), Movement (Kentel and Dobson, 2007), Emotional, Media, Financial (Gaspésie Literacy Council, 2021), Digital (Gilster, 1997), Artistic (Klein, 2019), Climate (Shwom et al., 2017), Political (Cassel and Lo, 1997), Information (Boh Podgornik et al., 2016), Science (Feinstein, 2011), Social (Arthur and Davison, 2000), Environmental, Ecological (Golley, 1998), Critical (Kalonji Rand, 2020), Ethical (Campbell and Hare, 1997), Legal (White, 1983), Interpersonal (Harder, 2011), Cultural (Hirsch, 1983), and of course Physical Literacy (Whitehead, 2001). This cursory search highlights that some examples are fully debated while others are simply appended without comment. It is also evident that there seems to be no common rationale for using the suffix. Where there is some detail, explanations include:

- the acquisition of knowledge and skills to function well in a particular culture (Hirsch, 1983)
- the enabling of effective participation in practices adopted in the parent society (Hirsch, 1983)
- attributing a meaning to all relations in one's social life (Ayyildiz-Durhan, 2020)
- “*the ability to access, understand, and use health information*” (Velardo, 2015, p. 385)
- being of value to the individual throughout life (Whitehead, 2010).

The recent range of areas adopting the term “literacy” highlights how the contemporary meaning is applicable within a variety of contexts concerned with the promotion of life skills, and/or developing human capabilities/capacity to live a full and flourishing life. There are also increasing examples wherein more than one “literacy” contributes to overall flourishing - such as an inclusive service approach in Scotland, wherein food literacy is coupled with physical literacy to respond to obesity (Gibson et al., 2019). The mixture of interpretations, however, still leaves literacy as a somewhat dynamic term. In itself, being dynamic is not an issue since concepts are not fixed, can be “multiple” (Hardman, 2019) and arguably, this aligns with ideas of ongoing development of capabilities/capacities. What is clear is that there is an observable desire to use the contemporary meaning of literacy to add value to existing areas and contexts. The contemporary understanding of literacy, however, as described in the opening introduction to this paper, is concerned with a much broader definition centring around the capability or capacity to productively and meaningfully interact with/in/through the world. The broader, richer notion of literacy encompasses more than reading/writing/language as discussed so far. Before embracing more recent thinking on literacy, we introduce the development of literacy with physical literacy.

### Literacy and Physical Literacy

Literacy, as a term, has also been used in conjunction with “physical” for over 100 years. Dudley (2018) and Cairney et al. (2019) both cite Officer Edward Maguire's (US Army Corp of Engineers) Professional Notes from 1884 as being one of the earliest recorded references. In the *Journal of Health and Physical Education* (1938), Sum et al. (2020) find reference to mental and physical literacy in the context of education and schooling. Around the same time, a letter by Strickland (1937, p. 10), recounts experiences of war, and “physical literacy” is used as simile. He parallels the misplaced enthusiasm of political leaders' celebrations of Armistice Day, with the surprise of traveling missionaries who experienced displays of “physical literacy” during meal preparations. The latter, likely experienced by Strickland as Officer in the British Army abroad, appears to be a singular use of the phrase. The display included ceremonial physical activity as part of a holistic appreciation for food, inherent to the particular indigenous culture witnessed by Strickland. Missionaries and political leaders are represented as being bewildered by respective displays. The use of “physical literacy” seems context dependent and not from sustained, considered understanding.

Acknowledging the contexts of the journey of physical literacy helps show how occasional and sporadic its use has been and what these were. For instance, many early uses seem to have had dualistic connotation, such as abilities related to health and fitness or adeptness in physical skill (Cairney et al., 2019). However, over the years the concept has broadened and, in some cases, has been seen as having a clear relationship to education as a whole and the holistic nature of each individual. Between 1927–1945 Cairney et al. (2019) suggest the term was used in response to post war physical health and concerns regarding a rapidly increasing mechanized society, as well as to highlight the danger of ignoring the physical aspects of pupils in schools. Later (circa 1958–1963), the term is used to champion the importance of fitness, physical and motor skills (Cahper, 1958 and Krug, 1960, both cited in Cairney et al., 2019).

The British Social Biology Council (1964) cited in Cairney et al. (2019) describes physical literacy as a type of communication, further explaining how it is the most basic form of communication, movement, mime, and creative manual work, with the emotional and verbal communication added thereafter. Interpretations from the late 1960s initially suggest a more holistic perspective and look more widely at human physical potential and terms such as “creative,” “inventive,” “sensitive” appear in the literature (Cairney et al., 2019, p. 81–82). However, toward the end of the 1970s there appears to have been a returned association of physical literacy with physical fitness (Cairney et al., 2019). These early uses and interpretations were used sporadically; it was not until Whitehead (2001) used the term in 2001, where a new meaning and interpretation would be the dominant, and contested, understanding for two decades.

Whitehead (2013) describes how when creating the concept of physical literacy, alternatives to the term “literacy” were also considered. Alternatives included “competence,” “ability,” and “skill.” However, Whitehead (2013) argues that physical competence, physical ability and physical skill leave the concept very much tied to pure physicality and using such terms may perpetuate dualistic perspectives. While physical competence forms a key element of physical literacy, the terms identified above would seem to place too much focus on the nature of human embodiment as a machine, object or an instrument and do not address the important role of embodiment-as-lived. Nor do these terms signal the interactive flux of embodiment as highlighted by philosophical schools of thought including those of monism, existentialism and phenomenology, which form Whitehead's (2001) foundations of physical literacy. This highlights how Whitehead (2010) carefully considered the selection of literacy as the most appropriate term to support and align with the philosophical foundations of the concept.

### The Evolution of the Contemporary Understanding of Physical Literacy

There was a significant period of time between Whitehead's doctoral thesis (1987) and her inaugural paper entitled “The Concept of Physical Literacy” (Whitehead, 2001). The time between 1987 and 2001 was devoted to presenting and developing the concept through wide-ranging seminars and conference presentations during which the concept took shape. In spite of writing on the centrality of embodiment (Whitehead, 1990), issues which Whitehead (1987) originally challenged in her PhD thesis, seem to remain today. The issues, in the context of physical education (PE) for Whitehead (1987), included the reduction of human beings into parts—usually as separate associations of mind and body; outcome over process; extrinsic rather than intrinsic worth of physical activity and; restricted perspectives on what constitutes a PE curriculum (that is, widespread focus on games and fitness). Quennerstedt et al. (2020) recently highlighted the “horrific narrative” that continues to interweave PE with poor health, arguably demonstrating the continuation of Whitehead's concerns with extrinsic and restrictive outcomes.

Whitehead was aware of the breadth of views and the controversies regarding the purposes and values of PE. She was also aware that the introduction of philosophy into the debate could prove a challenge. Thus, it is not surprising that the 2001 paper ends with a long list of questions, many of which are still unanswered. Interestingly, a question surrounding whether “literacy” is a more appropriate term than “mastery” or “competence” was included, as well as other questions such as whether Physical Literacy is a universal concept and is Physical Literacy an end state?

Since her (re)introduction of the construct physical literacy (Whitehead, 2001), the definition has been refined throughout the last two decades (Robinson et al., 2018). In part, Whitehead (2010) has worked to make clearer the philosophical ideas in her thesis: phenomenology, existentialism, monism and commitment to a holistic characterization of the human being. Further, Whitehead has responded to calls for empirical evidence which shows how the philosophy can be evidenced by practice, throughout the lives of individuals. For example, Whitehead (2019) has collected detailed evidence from around the world where physical literacy is demonstrably contributing to improving the lives of various populations, as well as being internationally relevant. We conjecture that this is also evidence of the fluidity of the concept.

Early debate on the concept of physical literacy picked up the notion of “literacy” either to highlight the cognitive demands of physical literacy (Roetert and Jefferies, 2014) or to align it to learning in linguistic literacy (Tremblay and Lloyd, 2010). The latter resulted in somewhat mechanistic and dualistic presentations focused on acquiring physical skills, often referred to as fundamental movement skills (FMS) (see Pot and van Hilvoorde, 2013; Afonso et al., 2014 for critique of this approach), or the ABCs of movement (agility, balance, and coordination). With the exception of Whitehead's (2010) book, the roots of literacy in the context of philosophy were not examined and hence not appreciated or understood. Whitehead's (2010) explanation that literacy was the enactment of human interaction with the world, was seldom referred to and was almost all but ignored.

The next period in the development of physical literacy saw a growing interest in the concept around the world, including Whitehead's (2019) book *Physical Literacy Across the World* and the production of a good many refereed papers and journal special editions. There is now a much livelier debate about the nature and extent of literacy within the context of physical literacy. Work in Canada with Indigenous educators (Nesdoly et al., 2021) and in New Zealand, where the Māori philosophy of Hauora underpins holistic wellbeing (Stevens et al., 2021) has proposed that literacy/interaction needs to address contact with other cultures, being mindful of spirituality and the relational. In Norway, it is argued that interaction with the environment, particularly nature, should have a higher profile (Lyngstad and Sæther, 2020).

Analysis of the research and policy discourse relating to physical literacy seems to demonstrate a re-call to early roots of physical literacy, as referred to above and detailed by Cairney et al. (2019). For example, following a critical explanation framework, Quennerstedt et al. (2020) suggest narratives of physical literacy concurrently hold “idealist” and “pragmatic” research and policy directions. The pragmatist narrative is tied very much to earlier manifestations of physical literacy as the remedy to public health concerns at various points in history, and indeed, current concerns with inactivity and rising obesity levels. Edwards et al. (2018) further point to the practical efforts of providing empirical evidence of whether physical literacy “makes” healthier individuals. This misses the centrality and nature of embodiment as essential to meaningful human flourishing (Durden-Myers et al., 2018). Indeed, efforts to unpin physical literacy from its philosophical roots changes the meaning of the concept in the lifelong and lifewide sense in which it is conceived by Whitehead (Young et al., 2020a).

The discussion and examples presented here, serve to highlight the multiple (re)interpretations of physical literacy as people “make sense” of the concept, in their own contexts. Currently, the International Physical Literacy Association [International Physical Literacy Association (IPLA), 2017] defines physical literacy as “the motivation, confidence, physical competence, knowledge, and understanding to value and take responsibility for engagement in physical activities for life.” In summary, the areas of contention over the last two decades appear to be represented by the following themes: fundamental movement skills as equivalent to physical literacy; synonymity with PE; omitting one of the four key elements of the construct as listed in the IPLA definition; seeking to measure physical literacy; physical literacy as providing gravitas when inserted into policy, such as for health, sport, or education policy (Dudley et al., 2017; Scott et al., 2021).

While it is of note that physical literacy is in no way a recently developed concept, it is worthy of mention that few of the earlier references appear to be related or founded on any clearly articulated philosophical principles that Whitehead's contemporary concept has at its foundation (Bailey, 2022). Physical literacy shares a similar development in history to that of literacy with contemporary interpretations moving on and away from associations with the ability to read and write, to arguably, a richer idea of the ability to productively interact and communicate with/in/through the world. These perspectives offer an interesting insight into the tensions emerging as the concept of physical literacy is more present in literature and in what we suggest could be a new phase of operationalisation and enaction. An appreciation of the aspirations of physical literacy alongside its current critique is essential in understanding how to best proceed in the promotion of physical activity for life through the nurturing of physical literacy. Certainly, the rationale and challenges for using the notion of “literacy” alongside “physical” is one of the key areas of discussion in the next section of this paper.

## Emerging Intersections

Literature within the field of physical literacy has reported difficulties and “*controversies*” (Whitehead, 2019, p. 13) in relation to the use, translation and understanding of the term “literacy” and its meaning(s). For example, despite careful consideration, the use of literacy within the concept has proved problematic. Physical literacy, and especially the literacy element, has been commonly interpreted as the ABCs (agility, balance and coordination) of movement and/or the ability to develop English literacy skills through physical activity (Durden-Myers, 2020). Both interpretations fall far from Whitehead's intended understanding of what is meant by physical literacy (Durden-Myers et al., 2020). Added to this confusion, the definition(s) of physical literacy have taken multiple forms in multiple countries worldwide, creating a chaotic situation (Edwards et al., 2017) which has given rise to a call for increased clarity and consensus.

These controversial areas can be regarded as intersections, a point where opinions, thoughts, theories or conceptualisations differ. Indeed, the differences occur under the same term, literacy. Here, Derrida's (1973, p. 284) term *differance* embraces the movement between what is different and what is *deferred* in meaning—an in-between, it “*recalls something like the middle voice*.” In effect, the historical conceptions of literacy and physical literacy are traceable also as present and future elements of it. The following discussion addresses some of the key intersections and areas of tension that exist in understanding literacy in/and physical literacy which may help develop clarity or if this is even a possibility (Martins et al., 2021).

## Discussion: Key Intersections and Areas of Tension

The discussion hereafter centers around key intersections and areas of tension associated with the use, interpretation and meaning of literacy in the context of physical literacy. Areas of tension are informed by the philosophy underpinning Whitehead's (2010) concept of physical literacy, as well as acknowledgment of Derrida's (1973) notion of *differance* and the introduction of the work of Barad (2013) on embracing multiple (re)presentations of phenomena and the effects of their differences. The discussion topics include: discourse, language, and interpretations of literacy; in/tangibility of literacy; capturing literacy; literacy as a process or a product; connotations of the terms literate and illiterate; neoliberalism and literacy, and finally literacy as learning.

### Discourse, Language, and Interpretations of Literacy

As noted above, interpretations manifest around the ways in which languages are constructed. Discourse analysis could highlight, for example, socio-political influence whereby physical literacy is written into policy because authors believe it adds gravitas of message to improve health (for example, Bailey, 2022, p. 171–172). The ongoing discussions about clarity of definition are in themselves co-constructors of discourses about literacy and physical literacy. For Derrida (1973) language, and effects of language, is in constant production to become fleetingly “present” to us. Young et al. (2021) observe and describe the physical literacy network of heterogeneous actors and their “voices” which exemplifies the controversies playing out in the field.

Physical literacy is tied to human nature which means it is applicable to all, (Whitehead, 2010). This universality means it has worldwide appeal which also gives rise to worldwide translations and interpretations. The work of translation, however, is not a simple case of exchanging one word for another (Davidov and Rappoport, 2009). Even the use of short phrases which appear to convey what is meant by, in this case, physical literacy will almost certainly result in a reduction of its full meaning. At the heart of physical literacy are its philosophical components, difficult to grasp in themselves (Edwards et al., 2017; Robinson et al., 2018) yet arguably, called for by practitioners, such as teachers of physical education in schools (Lundvall, 2015). Translating across languages is but one aspect to interpretation.

Further, stabilizing conceptual understanding of physical literacy does not support potential “here and now” uses of the term, in whichever context it is being used/applied (Young et al., 2020a). By here and now, we mean to suggest that whilst the concept is fluid, dynamic, flexible, multiple, it exists in its manifestations (Hardman, 2019). There may be multiple instances of physical literacy being repeated around the world, and these cannot be identical because of (again) multiple situations, interpretations and practices of “doings” of physical literacy (Barad, 2007). That said, there may be fundamental elements if something is claimed to be physical literacy, and these may resonate throughout its instantiations. One solution to issues of literal translation could be to provide a word list, perhaps in specific instances and languages, to denote other translations (Davidov and Rappoport, 2009). Given digital and technological capacities, this might be achievable in electronic documentation. Issues of literal translation are compounded by common interpretations of literacy as English literacy rather than its broader, richer contemporary meaning. As alluded to earlier, the former interpretation seems problematic.

What is clear is that physical literacy is not alone in its challenges with the interpretation of literacy worldwide. United Nations Educational, Scientific and Cultural Organisation (UNESCO) (2019) has used the term literacy for the past six decades within many programmes, with international reach. From its earlier inception literacy, as reading and writing, has morphed multiple times through UNESCO policy and research literature, much of which has been referred to above. Arguably, the use of the term literacy is gaining traction around the world and is doing so in multiple. For example, on the theme of Literacy, United Nations Educational, Scientific and Cultural Organisation (UNESCO) (2019) states that:

*Beyond its conventional concept as a set of reading, writing and counting skills, literacy is now understood as a means of identification, understanding, interpretation, creation, and communication in an increasingly digital, text-mediated, information-rich and fast-changing world*.

At policy, development agenda and implementation levels, there remains the central drive to improve “basic reading and writing skills” [Global Alliance for Literacy, United Nations Educational, Scientific and Cultural Organisation (UNESCO), 2019]. Thus, it seems in practice, “conventional” literacy does still dominate. UNESCO has also referred to physical literacy within its documentation. Physical literacy was referred to in the Quality Physical Education framework in 2015 [United Nations Educational, Scientific and Cultural Organisation (UNESCO), 2015] and continues to feature in the updated 2021 version of this document [United Nations Educational, Scientific and Cultural Organisation (UNESCO), 2021]. This does signal that physical literacy continues to be important despite challenges of discourse, language and interpretation around the world.

### In/Tangibility of Literacy

Building from the challenges of multiple interpretations of literacy is the notion or idea that literacy is not a fully tangible concept. From the earliest scholars writing on the history of literacy to current literature, the term literacy remains untethered, a ‘will o' the wisp' character. Its application, ironically through literature, to an increasing number of society's practices reflects a simultaneous increase beyond text-based associations. “Multiliteracies” or “alternative literacies” are terms discussed by Provenzo et al. (2011), drawing from The New London Group (1996), which capture the notion of dynamism and more-than “alphabet” based literacy. They suggest that, for example, visual ways of communicating have, at times, been more powerfully communicative than text-based “conventional” literacies—such as cave writings and hieroglyphics. In their edited work, Provenzo et al. (2011) share work of quilters, rappers, animal lovers, to highlight alternative literacies and the work they do in communicating and empowering often unheard or misunderstood voices.

The variety of methods, intangible as some might appear, need not be hierarchical capacities for interaction or communication. For instance, shorthand methods of producing the written word do not necessarily preclude conventional literacy development (Drouin and Davis, 2009). Embodiment is afforded multiple avenues for communicating and developing human understanding and reading with/in/through the world. As with literacy, physical literacy is similarly one of multiple ways of playing and communicating between and within cultures. Living with multiples and in a constant motion, seems to underpin the elusive search for concrete meaning of physical literacy.

Physical literacy reveals and unravels through experienced motility. *Differance* (Derrida, 1973; Baugh, 1997) in the context of physical literacy, identifies an in-between space of non-identical-sameness (non-identity). Physical↔literacy in constant motion articulates a *middle voice* as it records the nature of passivity and activity. In-flight, physical↔literacy positions itself in the in-between spaces (spoken and written word), neither captured nor absolute. Examples of this can be found when cooking, playing a musical instrument or sending a ball and moving to space. Therefore, physical↔literacy is a living library that documents thoughts, interactions, and musings through chaptered experiences (Dhillon, 2018). Yet while this understanding is known within the field of physical literacy there remain calls to capture, measure and assess physical literacy in order to be able to legitimately evidence and embrace the concept in a variety of sectors (Nesdoly et al., 2021).

### Capturing Literacy

Seeking clarity of terminology seems not necessarily to mean identifying, observing, measuring or “capturing” literacy. Rather, as we discuss in the previous section, concepts are fluid and not bound by time and space (Hardman, 2019). Therefore, the concept of physical literacy is fluid. In clarifying physical literacy we seek not to represent—like two mirrors epistemologically reflecting knowledge back and forth where nothing more is seen (Barad, 2003)—but to act more like the waves of the ocean. “Diffraction” comes from the world of physics and behaviors of matter, either as particle in linear fashion or splitting or indeed, at once “cutting together-apart” (Barad, 2013). The waves of the seas, constantly in flux, transversing whilst folding in on one another, are still recognizable as the seas. The singular concept of the sea is synonymous with the multiplicity of seas all at once, continuously, thus always becoming (Hardman, 2019). Physical↔literacy may then be understood at once as a concept and as multiple. Barad's (2013) notion of diffraction brings to life Derrida's ideas of past-present-future and non-linearity of time, hence bringing theorists together in this paper.

For Derrida (1973), the signified concept is never present in itself. Further, one person's physical↔literacy journey will not be the same as the next, as is understood phenomenologically. The realization that one's past-present-future physical activity *is* the phenomenon physical literacy, provides a challenge to linear ideas about “*classical ontology*” (Barad, 2013, p. 28). A person's relationship with physical activity is better compared visually to the ever-changing shape of a flock of birds rather than to a line on a graph. Again, while this is widely accepted within the field of physical↔literacy, there remains a demand for evidence and tools to capture the “effects” or “outcomes” of physical literacy “programmes” (Green et al., 2018; Stevens et al., 2021; Bailey, 2022). As we discuss, fully capturing physical↔literacy is simply not possible to do. It is possible to capture snapshots, moments, reflections and perceptions relating to movement experiences that can contribute toward creating a picture, identifying characteristics and principles that support the nurturing of physical↔literacy. But in this instance the interpretation of, and the construction of “legitimate knowledge” from these fleeting moments will be contextually bound. Kirk (1992) articulates this by describing how “legitimate knowledge” is not fixed, but is instead constantly in process, shaped by social, political, and cultural, as well as wider forces; “legitimate knowledge” is also not politically nor culturally neutral. Reflecting upon Kirk (1992) and Croston and Hills (2017) also argue that actions when making decisions on “legitimate knowledge” are therefore rarely accidental; they have an origin, a history and are almost always inevitably constrained by prevailing political ideologies (Evans and Penney, 2008). Therefore, a physical↔literacy informed approach is an interesting debate that links well to the next section around whether physical↔literacy can be considered a process or a product or as we suggest somewhere in between, and indeed the previous section around the intangibly of literacy. Physical literacy is conclusively in flux and as such so too are (re)presentations of physical literacy (Barad, 2013).

### Literacy as a Process or a Product

There is current academic debate surrounding whether physical literacy is a process or a product. Notably, the process or product debate is prevalent in literature aiming to understand how to assess physical literacy, particularly in the context of schooling. Young et al. (2020b) describe practices within health and physical education assessment, which have been critiqued for having an overly technical, performative, or product focus. Product focus results in narrowing of, and objectively assessing, fundamental motor skills or fitness, combined with an interest in student management, as opposed to student learning (Hay, 2006; Penney et al., 2009; López-Pastor et al., 2013).

Notably, existing scholarship suggests that a range of perspectives exist toward assessment of physical literacy (Edwards et al., 2017; Green et al., 2018; Whitehead, 2019; Goss et al., 2021). In the context of schooling, some scholars acknowledge that positioning physical literacy within Health/Physical Education (H/PE) means that it must always be understood in relation to curriculum, pedagogy, and assessment of and for learning (for example, Hyndman and Pill, 2018). Others, however, prioritize the assessment of often isolated physical movement and skill competency (Cools et al., 2010). The process or product debate is also found when using and referring to the terms and notions of literate, illiterate and physically educated.

### Connotations of the Term Literate and Illiterate

Roetert and MacDonald (2015) argue that the term “physically educated” implies a finished product, so too can be said for the term “physically literate” which may encourage a view that it can be “achieved” or “accomplished” (Quennerstedt et al., 2020). Instead, Whitehead (2013) argues that physical literacy connotes an ongoing process according to an individual's interests, past experiences and future opportunities. Physical literacy can be considered therefore as an ongoing lifelong process or journey (Whitehead, 2010).

Whilst acknowledging the logic that since all human beings are moving beings, there is always a level of physical capability, Whitehead (2010) does make clear that a person can be physically illiterate. Such an individual would have no desire to engage in physical activity beyond functional everyday movement, might use transport for short journeys rather than walk, for example. This person will not have confidence in her/his physical activity capabilities since physical competence has not been developed and extended. In turn this might manifest immature movement patterns or poor physiological health. Presenting the potential for illiteracy does infer a dichotomous notion of literacy and implication that the illiterates require “*treatment to remedy their malady*” (Maposa and Wassermann, 2009, p. 43).

Rather than adopt this quantitative approach to literacies, a pluralist perspective would allow encounters in non-linear, processual ways (Maposa and Wassermann, 2009; Edwards et al., 2017). In doing so, this may increase opportunities for inclusivity, cultural relevance (Stevens et al., 2021) and recognition of holistic indigenous approaches (Nesdoly et al., 2021). Two points are worth mentioning here. First, the pluralist approach presents a challenge to the climate of accountability which constantly reduces human beings to parts. Second, traversing multiple literacies (capabilities) in a non-linear way also aligns better with constructivist approaches to education eschewing fixed developmental progression. For example, the individual mentioned above, who is not physically active beyond functional everyday movement, may take up a new physical activity. Each experience (of moving and moving for everyday function) seems to exemplify a continuum of physical literacy rather than a “starting point” of being physically illiterate.

To be clear, using the terms literate and/or illiterate is not necessarily helpful predominantly because both terms are reductionist and possessive in nature—that is, a person has or does not have, which is not congruent with the literature stating that all have the individual endowment and potential to make progress on their physical literacy journeys. In essence, all individuals are embodied and on continual journeys of discovery in relation to their own physical literacy. Implications of this in practice would include being less reliant on reductionist judgements made by others reducing a person's lived experience and embodied potential to just a mere number or adjective. Additionally, more sensitive consideration of the cultural-discursive language used when talking about movement experiences is essential, especially when privileging certain types of activities (indigenizing/de-colonizing the curriculum) or reinforcing stereotypical desirable moving bodies, which can be elitist and exclusionary in nature. Instead, we advocate that the deliberate act of reflection, by the participant independently or supported by others, is the valuable learning taking place, and helps ensure that inclusion is centrally informing an embracing of wide-ranging embodied lived experiences.

### Neoliberalism and Literacy

Our argument thus far has called for acceptance of a broad notion of (physical) literacy, running against the prevalence of calls for definition clarity. In refining the concept of literacy, it becomes restricted, and tightening in this way ensures the term is exclusive (Sims, 2017). Understood like this, literacy fits neoliberalist desires to maintain a healthy workforce, wherein physical activity and physical education have been frequently included in discussions (Kirk, 2010; Quennerstedt et al., 2020). It could be argued that as a politico-cultural-economic tool literacy (and we would argue physical literacy) becomes the preserve of the few, in essence an “*elitist*” construct (Maposa and Wassermann, 2009, p. 42). In essence physical literacy in a neoliberal society can become a knowledge commodity which then moves away from the inclusive and human flourishing philosophical roots (Durden-Myers et al., 2018) with it becoming the preserve of the privileged few. Young et al. (2020a) have moved the physical literacy research field on in this regard, by presenting how a “ladder of abstraction” can present a more pluralistic approach to the conceptual understanding and (re)presentation of physical literacy. On the other hand, if literacy is embraced as inclusive where individuals are encouraged and empowered to communicate in different environments and in multiple ways, we must also embrace multiple meaning(s), doing(s) and knowing(s) (Nesdoly et al., 2021; Stevens et al., 2021). As an example from “conventional” literacy, the use of shorthand “text speak” or emoticons (Drouin and Davis, 2009), or presentation of academic research in visual form such as comics (Sousanis, 2015), provide access for more rather than less people. At the same time these examples, arguably, provide challenges to the privilege of the written word whilst moving beyond neoliberal restrictions.

Physical literacy is currently discussed in academic literature, as well as being embraced in policy, such as Canada's Physical Literacy Consensus Statement (2015) or some health, education and sport policy (Dudley et al., 2017; Scott et al., 2021). Our paper calls for this to be changed. If physical literacy is to realize its full potential, more inclusive ways of embracing literacy and physical literacy are required. Whilst acknowledging the limits of the Canadian Consensus process, Tremblay and Longmuir (2017) suggest multi-sector engagement. Thus, we would add indigenous populations, actors from transport and urban planning, and representation from all age groups to the conversation, with the aim of increasing opportunities for all to flourish (Sims, 2017). After all, if the concept is inclusive and is indeed for all, it needs acknowledged as permeating and transcending many areas of life, not just limited to, for example, academia and education (here, one could read “schooling”).

### Literacy as Learning

Literacy as interaction and communication represents the human individual as forever searching for meaning, resolving dissonance, finding balance and harmony. It is an expression of intentionality—the endless urge to relate, to affirm our being, to make sense of the world and ourselves (Merleau Ponty, 1945/2012). The interaction/communication has developed affordances to aid effective relationships with the world (Durden-Myers et al., 2021). Literacy could be seen as the foundation of life—using capabilities to create ourselves as we enact a lifetime of dancing with the world (Dhillon, 2018; Dhillon and Ulmer, 2021). As such, literacy is learning, but in the widest sense of the word (not cognition), learning from previous experiences, encounters at every level from precontemplation, contemplation and tacit knowledge.

A Visual Biography (Dhillon, 2018; Dhillon and Ulmer, 2021) documents human experiences at dynamic junctures, exhibited through multifaceted timeline trajectories (young-senior, open-close, new-old). This assemblage meanders through motility, accessing temporal-spatial dimensions, both visible and invisible (wind, water, walking, using a wheelchair, gravity). Such landscapes proliferate the “cyclical” body as lived-living-lived. Providing temporal-spatial opportunities to meaning-making moments creates opportunities to explore and solidify an ever-evolving interaction (within the activity). Meaning-making questions creativity in the context of immersive experiences. The reaction to scoring a football goal or having legitimate tackles penalized after the fact is incubated knowledge, a lived experience. Often, we (as spectators) will experience footballers celebrating goals only to realize that the goal was ruled offside (the evolution of Video Assistant Referees). Living-lived body reality is captured at these poignant moments, shifting the contextualized identity and composition of the ideals contextualized in football. At this juncture, creativity, assemblage, space and place are minutely compromised (continuum) as they begin to shift. Collectively, the intricate play of spatial/temporal trajectories contribute to the “*circle of wellness*” (Cardinal and Pepler, 2021, p. 5) further questioning community narratives.

The Body as a Knowledge Incubator™ (Biso, 2020; Durden-Myers et al., 2021) can be defined as a perpetual encoding of motility. Physical literacy informs and is informed in multiplicity. The literacy of the body is fluid, tangible, intangible, and structureless allowing beings to roam (Durden-Myers et al., 2021). Physical↔literacy musings evolve in a lived-world and can be located in tangible examples such as within Nature (seasons, colors, shapes), which are being-present-fleeting. Innately, such musings are neither present, being or absent. Tangibly, we try to capture/understand physical literacy when we experience activity/passivity. For example, we may change our flight trajectories when experiencing tail/headwind and account for wet/dry surfaces when playing sports. We have moments of dissonance that call forth our innate desire to seek harmony, to propel knowledge and to discover new action-potentialities. This can be seen in the learning of a new skill, refining a dance routine, or evaluating our performances. Educators, coaches, mentors, inter alia, have an opportunity to supportively craft and provide enabling environments that draw forth this opportunity to (re)discover our potentialities (Durden-Myers et al., 2021).

As physically active humans, we manipulate and explore pathways that are ever-present, being-presented. For example, a figure skater may seek to deliver an *authentic* routine to hit a perfect score (10). Snowboarders completing a half-pipe event at the Olympics may complete specific actions to extend their creative prowess. These are tangible, meaningful endeavors in which the *Body* absorbs, assembles and takes flight, again and again, in relations with others, with things in the environment, assemblage/bringing-together (Derrida, 1973). Physical↔literacy, therefore, becomes a focal point. A place of assembly in which humans explore and create through space, over time (motility). This progressive movement is an expression of lived-living-lived motility. By recording tensions through expressions, The Body as a Knowledge Incubator™ allows the *physical* to become both a metaphor and a mechanism. Place making and attachment are points of tension (incubated/yet to be incubated knowledge).

**Figure 1:**
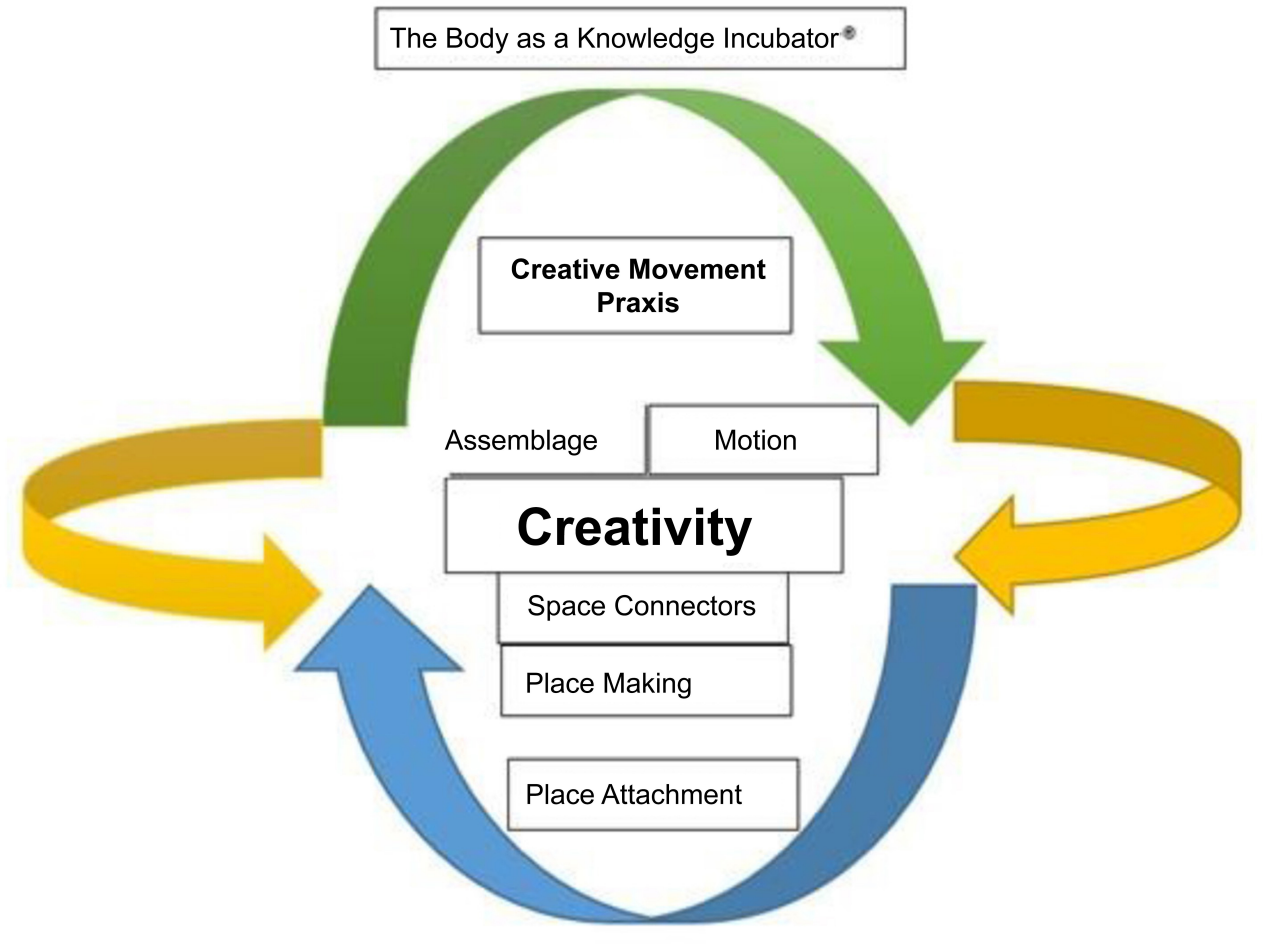
The Body as Knowledge Incubator™. Dhillon (2018), Biso (2020), and Dhillon and Ulmer (2021).

The terms used in Figure 1 are defined in Appendix 1. A *Body*, questioning/able is a prevalent connector and a pathway (evolving) negotiating spaces—creative praxis. At this juncture, praxis (action-reflection) records individuals' innate creative endeavors as they strive toward understanding their environments or those they are propelled to/away. For example, an Olympian may hit a perfect score on home turf at National competitions yet cannot replicate the same routine at the Olympics. Over time, personalized motility (signatures) can be a product of knowledge that seek(s) to connect with hybrid, virtual, local, and unfamiliar spaces. This harmony is a process/reaction/synergy that interacts/connects/creates an equilibrium between the living/lived Body (Durden-Myers et al., 2020). Therefore, the tensions which exist are the existence of harmony, the in-between and the gaps (Baugh, 1997). Physical↔literacy in all forms of expressed motility (force, flight, playing a musical instrument, cooking, activities in general and sports) is discovered and yet to be discovered.

Physical↔literacy is a site of discovery, proliferated by lived realities. Lived reality—middle *voice* becomes a place of contention (Derrida's *differance*) and thus a place of learning. Within the context of The Body as a Knowledge Incubator™, middle *voice* is silent, salient, robust and articulative. Middle *voice* meanders in-between as spoken, performed and/or written literacy. As a Visual Biography it teaches us about ourselves, and others and our place in community. It narrates our storied lives.

## Conclusion

In this discussion paper we have tried to unpack the complicated nature of literacy in its physical context. Framing Physical Literacy in its current operational state requires acknowledging its competing narratives within the contexts of Physical Education, Physical Activity, Dance, Sport and Recreation. By unpacking the nature of physical literacy, we discuss the variabilities and entanglement, the in-between spaces. The introduction of The Body as Knowledge Incubator™ purposefully (re)orients the reader to the tangible. Two tangible examples of incubated knowledge include: (1) the body is forever recording interactions that are both meaningful/less to the individual. As the body ages, literacy records the necessity for lifelong learning or understanding of our physical interactions; (2) emancipatory language also provide the body digestible knowledge especially in volatile social and political conditions. Community languages/dialects, student language and dominant languages question conscientization (Freire and Macedo, 1987).

Consequently, we invite readers to introspectively reflect on their physical literacy whilst reading this discussion paper. Known as entry points, further investigative thought invites the audience to journey to the cusp of what Derrida (1973) and Barad (2013) describe as newness (Thiel and Hofsess, 2020). Newness lends itself to the concept of multiplicities, where trajectories of physical literacy are evolving (such as the definition), thus physical literacy is orbiting (not linear). As a reader, participant, researcher, or teacher, we ask you to think through practices that harness the true nature of literacy in the physical world. Locating the necessity of philosophizing Physical Literacy may provide new(er) terrain to better understand literacy.

Literacy is the abilities or capacities of an individual, in a given context, to be able to observe, understand, interact and respond to environments effectively. In other words, the life skills that are formed through literacy provide a critical feature of what it means to be enacting literacy but it must also be relevant and applicable to the individual's context and/or environment. Physical literacy is not unique but instead sits alongside other (human) capabilities. Sadly, in our dualistic world this is not always appreciated. We believe physical literacy is the key literacy on account of our embodied and physical manifestation in the world. Often Physical Literacy is the literacy through which other literacies have to pass. Physical literacy aims to invigorate and elevate the importance of our embodied selves. Through physical activity individuals can not only nurture their own physical literacy but also contribute toward our holistic literacy(ies) that helps us navigate, connect and make sense of ourselves, others and the world around us.

Our intention within this paper was to provoke thought and highlight the dissonance found at intersections, not to land on one side or the other, but instead to embrace the in-between space, emphasizing this as fertile ground where sense-making and meaning-making can flourish through the embracing of *difference* (Derrida, 1973), and by challenging the singular representations of concepts (Barad, 2013). We hope that our discussion has shown that literacy is a fluid term, meanings are woven into the fabrics around the world in multiple ways. Different languages add specific translations in addition to complexities of cultural and individual efforts to understand the multiple interpretations of literacy and physical literacy. Phenomenology informs physical literacy, thus humans are constantly becoming as they are co-created whilst interacting with the world. We suggest that physical literacy as interwoven with the individual's movement with/in/through the world is the embodied way in which co-creation is explored, manipulated, developed, challenged and extended. We hope that this paper provokes others in embracing the in-between space; entry points of nonidentity (sameness) and challenges the singular representations of concepts, particularly focusing on multi-modal literacies and inclusive methodologies and pedagogies. We look forward to continuing to observe the ever-changing shape, direction and dance of the flock of birds that is physical literacy.

## Author Contributions

ED-M conceptualized the article content. All authors discussed content and wrote and edited the work. All authors contributed to the article and approved the submitted version.

## Conflict of Interest

The authors declare that the research was conducted in the absence of any commercial or financial relationships that could be construed as a potential conflict of interest.

## Publisher's Note

All claims expressed in this article are solely those of the authors and do not necessarily represent those of their affiliated organizations, or those of the publisher, the editors and the reviewers. Any product that may be evaluated in this article, or claim that may be made by its manufacturer, is not guaranteed or endorsed by the publisher.
