# Supplementary material for: Exploring the Notion of Literacy Within Physical Literacy: A Discussion Paper
Source: Front Sports Act Living. 2022 May 3;4:853247. doi: 10.3389/fspor.2022.853247 (PMC9110965; doi:10.3389/fspor.2022.853247)
Supplement: Supplementary file 1 [file Table_1.pdf]

Appendix 1: Creative Movement Praxis Definition of Terms

Visual Biography	A methodological component of phenomenology capturing manifestations of unit of experiences through movement and photography.
Creativity	Continuum-temporal-rhythm embodied by self.
Assemblage	A multiplicity of 'events,' collection of 'things' or 'actors' held together (relation).
Motion/Motility	Intensive points of an infinite number of points at varying singularities.
Space Connectors	A plurality of forces acting and affected by incubated knowledge within the context of a lived-living body.
Place Making	Virtual multiplicities or intensities that consistently document accessed spaces that are localized by personhood.
Place Attachment	A process of differentiation within the context of self-determination. A durable and robust passage of connection both virtual to actual within a thing.

Glossary (Baugh, 1997; Dhillon, 2018; Dhillon & Ulmer, 2021)
